# Analysis of hemisphere-dependent effects of unilateral intrastriatal injection of α-synuclein pre-formed fibrils on mitochondrial protein levels, dynamics, and function

**DOI:** 10.1186/s40478-022-01374-z

**Published:** 2022-05-23

**Authors:** Rose B. Creed, Adeel A. Memon, Sindhu P. Komaragiri, Sandeep K. Barodia, Matthew S. Goldberg

**Affiliations:** 1 Center for Neurodegeneration and Experimental Therapeutics, The University of Alabama at Birmingham, Birmingham, AL 35294 USA; 2 Department of Neurology, The University of Alabama at Birmingham, Birmingham, AL 35294 USA; 3 Department of Neurobiology, The University of Alabama at Birmingham, Birmingham, AL 35294 USA; 4 Neuroengineering Ph.D. Program, The University of Alabama at Birmingham, Birmingham, AL 35294 USA

**Keywords:** Parkinson’s disease, Synuclein, Pre-formed fibrils, Lewy bodies, Aggregation, Mitochondria

## Abstract

Genetic and neuropathological evidence strongly implicates aberrant forms of α-synuclein in neurodegeneration. Antibodies specific for α-synuclein phosphorylated at serine 129 (pS129) are selective for the pathological protein aggregates that are characteristic of Parkinson’s disease (PD) and other synucleinopathies, such as dementia with Lewy bodies (DLB) and multiple system atrophy (MSA). Although the etiology of most synucleinopathies remains uncertain, a large body of evidence points to mitochondrial dysfunction. The recent development of animal models based on intracranial injection of α-synuclein pre-formed fibrils (PFFs) has provided a valuable experimental system in which to study the spread and neurotoxicity of α-synuclein aggregates, yet the effects of PFF-induced protein aggregates on mitochondrial function and dynamics have not been rigorously examined in vivo. To help fill this knowledge gap, we injected the striatum of mice unilaterally with well-characterized small length (< 30 nm) PFFs or monomeric α-synuclein control and measured the distribution and extent of pS129 α-synuclein-immunoreactive aggregates, the loss of tyrosine hydroxylase-immunoreactive neurons in the substantia nigra, the abundance of mitochondrial proteins, and the activity of mitochondrial respiratory chain components at 3 months and 6 months post injection. Intrastriatal injection of small length PFFs, but not monomeric α-synuclein control, induced robust pS129 α-synuclein immunoreactive inclusions in the cortex, ventral midbrain, and striatum, as well as in rarely reported brain regions, such as the hippocampus, as early as 3 months post injection. Significant loss of nigral tyrosine hydroxylase-immunoreactive neurons was observed in the PFF-injected hemisphere at 3 months and 6 months post injection. The unilateral striatal injection of small length PFFs also caused hemisphere-dependent and treatment-dependent changes in the cortical levels of mitochondrial proteins such as VDAC1, COX-IV, and DRP-1, as well as functional changes in mitochondrial complex I activity in the contralateral striatum. Together, these data demonstrate that intrastriatal injection of mice with small length PFFs induces extensive bilateral protein aggregates, significant unilateral nigral cell loss, and altered contralateral levels of mitochondrial proteins and respiratory chain activity. Our data suggest this animal model may be useful for studying the role of mitochondrial dysfunction in α-synucleinopathies, for studying the hemisphere-dependent effects of α-synuclein aggregates, and for testing neuroprotective therapies that target mitochondrial dysfunction and protein aggregation.

## Introduction

Protein aggregation is a pathological hallmark of many neurodegenerative diseases. α-synucleinopathies are a class of neurodegenerative diseases defined by the presence of aggregated forms of the protein α-synuclein. These include Parkinson’s disease (PD) and Lewy Body Dementia (LBD), which show neuronal synuclein pathology termed Lewy bodies and Lewy neurites; and Multiple System Atrophy (MSA), which is characterized by astroglial α-synuclein inclusions. In PD, α-synuclein pathology is hypothesized to drive the loss of dopaminergic neurons of the substantia nigra pars compacta (SNc) which are necessary for normal movement [6]. Identification of PD-linked mutations in the α-synuclein encoding *SNCA* gene [2, 10, 48, 56] has led to the development of α-synuclein transgenic and viral overexpression animal models to study both the normal function of α-synuclein and the mechanisms by which α-synuclein aggregation causes neurodegeneration. Despite many studies using a variety of animal models of α-synucleinopathies, our understanding of the precise mechanisms by which α-synuclein aggregation causes or contributes to disease remains limited.

There is substantial evidence implicating mitochondrial dysfunction in α-synuclein-associated neurodegeneration. α-Synuclein can biophysically interact with mitochondria under physiological conditions [34]. Analysis of individual midbrain neurons laser-captured from postmortem PD brain tissue revealed significantly greater mitochondrial DNA mutations in neurons with Lewy bodies compared to neurons without α-synuclein pathology [43]. Abnormalities in components of the mitochondrial electron transport chain have been described in MSA, LBD, and PD [27, 29, 41, 42], with reductions in complex I activity prominent in PD [21, 33]. Furthermore, individuals with mitochondrial disease are more likely to develop Lewy pathology [18]. Many cell, animal, and postmortem studies have shown that mitochondrial dysfunction promotes or exacerbates α-synuclein pathology [9, 11, 12, 15, 30]. Determining whether and how α-synuclein pathology affects mitochondrial function, dynamics, and abundance in the context of nigral dopamine neuron loss is important for informing therapeutic efforts that target α-synuclein and mitochondrial health.

Numerous viral vector and transgenic animal models overexpressing α-synuclein with and without disease-linked mutations have been used to study the pathophysiology of α-synucleinopathies [20, 32, 65, 66]. However, these models vary considerably with respect to neuropathological and behavioral phenotypes, possibly due to differences in the cell type, brain region or level of transgene expression. The recent development of the α-synuclein pre-formed fibril (PFF) model of α-synuclein aggregation and neurodegeneration provides a potentially more uniform and robust experimental system in which to study pathogenic mechanisms [35]. In this model, α-synuclein pathology is seeded by corruption of endogenous α-synuclein species by intracranial injection of fragmented pre-formed fibrils composed of recombinant α-synuclein, which initiates the formation of α-synuclein aggregates both near the site of injection and in brain regions with neurons that project to that site [36, 67, 68]. The PFF animal model is gaining widespread use for studying the spread of α-synuclein pathology across brain regions and for exploring mechanisms of neuronal toxicity, particularly degeneration of SNc neurons [1, 45–47, 55]. Intracranial injection of mice or rats with α-synuclein PFFs reliably induces the conversion of endogenous α-synuclein into visible brain inclusions that spread with time and are immunoreactive for α-synuclein phosphorylated at serine 129, which is a relatively selective marker of synuclein pathology in human postmortem brains and animal models [24]. In vivo and in vitro studies using α-synuclein PFFs have provided valuable insights into α-synuclein-induced neuroinflammation [16, 28] and changes in synaptic transmission and dendritic morphology [17, 23, 71]. However, little is known about the effects of α-synuclein preformed fibrils on mouse brain mitochondria upon intrastriatal injection. Because mitochondrial dysfunction and α-synuclein aggregation are both strongly implicated in the pathogenesis of PD, as well as other α-synucleinopathies, it is important to rigorously examine the effects of α-synuclein PFF-induced aggregation on mitochondrial abundance and function in vivo*.* Therefore, in addition to neuropathological characterization, we measured mitochondrial respiratory chain complex activity and levels of proteins involved in mitochondrial function and dynamics in brain homogenates from the injected and uninjected hemispheres of mice 3 and 6 months after unilateral intrastriatal injection of well-characterized α-synuclein PFFs.

## Materials and methods

### Animals

All experiments were conducted after review and approval by the University of Alabama at Birmingham Institutional Care and Use Committee. Approximately 10-week-old male and female wild-type C57BL/6 J mice were obtained from the Jackson Laboratory and allowed to acclimate at least 2 weeks but not more than 5 weeks before surgery. Animals were maintained on a 12-h light/dark cycle and allowed food and water ad libitum.

### Fibril generation, sonication, and characterization of PFFs

**Fig. 1:**
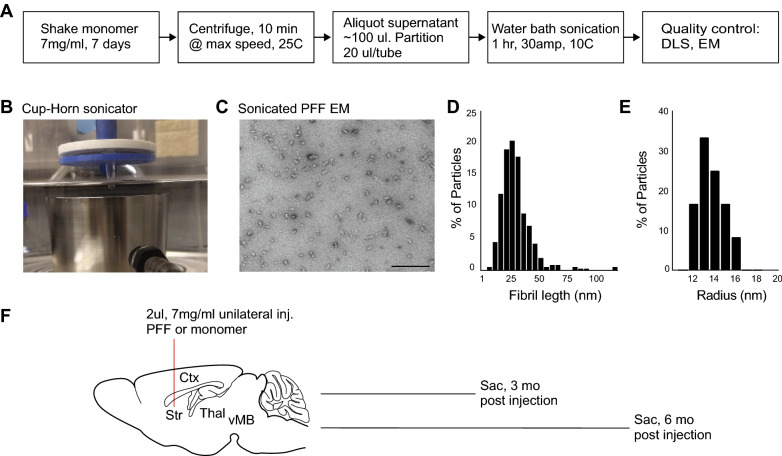
Fibril generation, quality control, and experimental timeline. **A** Flow chart of procedure used for fibril generation and quality control analysis. **B** Cup-horn sonicator used to sonicate fibrils. **C** Electron micrograph of sonicated PFFs diluted 1:300 in PBS and spotted on a formvar-carbon coated EM grid. Scale bar is 100 nm. **D** Histogram showing the length distribution of sonicated particles measured from the EM micrographs. Fibril lengths were generally uniform with a median length of 29.5 nm. **E** Histogram of radii of sonicated PFFs measured via dynamic light scattering (median 13.5 nm). **F** Schematic of intrastriatal injection of 2 μl of either monomer or PFFs followed by euthanasia and harvesting brain for analysis either 3 or 6 months post injection. Ctx: Cortex, Str: Striatum, Thal: Thalamus, vMB: Ventral Midbrain

Recombinant full length unmodified mouse α-synuclein monomer (gift from Andrew West and Laura Volpicelli-Daley) was expressed in *E. coli* and purified as previously described [67] with removal of lipopolysaccharide using a high-capacity Endotoxin Removal spin column (Pierce). Residual endotoxin levels were determined to be 0.017 Endotoxin Units/μg protein using an LAL chromogenic endotoxin quantification kit (Pierce). To generate fibrils, 7 mg/ml monomeric α-synuclein was shaken in an Eppendorf tube at 700 RPM for 7 days at 37 °C in 50 mM Tris pH 7.4 plus 150 mM KCl. The resulting cloudy solution of fibrils was centrifuged for 10 min at 16,000 × g at 25 °C. The clear supernatant was removed and discarded, leaving a solution of highly enriched fibrils as starting material. This was aliquoted into 20 µl per PCR tube, then immediately sonicated for 1 h continuously (with no on/off cycles) at 30% amplitude using a Qsonica model Q700 cup horn sonicator with only the 20 µl of fibrils just under the surface of a 10 °C circulating water bath (Fig. 1B). The solution was no longer cloudy when the sonication was complete. The 20 µl aliquots of sonicated fibrils were pooled together, and the protein concentration was measured by the absorbance at 280 nm in a nanodrop spectrophotometer using an extinction coefficient of 7450 M^−1^ cm^−1^. PFFs were diluted to 0.1 mg/ml in PBS and analyzed by dynamic light scattering (DLS) to calculate the PFF hydrodynamic radius from the time-dependent fluctuations in scattered light intensity at 25 °C using a Wyatt Technology DynaPro NanoStar instrument with DYNAMICS software. PFFs were also analyzed by electron microscopy by diluting PFFs to 0.3 μg/ml with PBS and spotting 3 μl on glow discharged 400 mesh copper grids, negatively staining with 1% uranyl acetate and imaging with a Tecnai F20 electron microscope at 42,000x. ImageJ was used to measure the length of all particles in 5 EM images (> 300 particles). Following sonication, PFFs were stored at 4 °C until completion of the surgeries, within 2 weeks.

### Surgeries

Mice were anesthetized with vaporized isoflurane and fitted to a stereotaxic frame (David Kopf). All injections were targeted to the right dorsal striatum at the following coordinates measured from bregma: A/P + 1.0, M/L − 2.0, D/V − 3.2. 2 ul of 7 mg/ml α-synuclein PFFs or monomer was injected at a rate of 0.5 ul/min. The syringe was held in place for an additional 5 min to ensure that all the solution was dispensed and dissipated into the brain. Incisions were closed by application of 3M Vetbond (MMM1469) and mice were returned to their home cages. At 3 and 6 months post injection, separate cohorts of mice were euthanized, and the brains were rapidly harvested and dissected on an ice-cold glass petri dish. The caudal half (containing the SNc) was post-fixed for 24 h in phosphate-buffered 10% formalin (Fisher SF100-4) at 4 °C, then transferred to 30% sucrose in PBS at 4 °C for at least 3 days for cryoprotection prior to sectioning and staining. The rostral half (containing the striatum) was microdissected and flash frozen for mitochondrial respiratory chain activity assays and for western analysis. At each time point, subsets of mice were deeply anesthetized with isoflurane and transcardially perfused with PBS. Entire brains were removed, post-fixed, and cryoprotected before sectioning and staining for immunofluorescence.

### Mitochondrial electron transport chain activity assays

Striatum samples were homogenized using a Wheaton mortar and pestle in 20:1 (volume/weight) ice cold mitochondrial isolation buffer (100 mM KCl, 50 mM Tris–HCl, 1 mM Na-ATP, 5 mM MgSO4, 0.1 mM EGTA, 0.2% BSA, pH 7.4, supplemented with Roche mini-complete protease inhibitor cocktail). Crude homogenates were centrifuged 10 min at 600 × g 4 °C. The supernatants were transferred to clean ice-cold tubes and centrifuged for 10 min at 10,000 × g 4 °C. The mitochondria enriched pellets were resuspended in 50 ul mitochondrial isolation buffer and protein content was measured using a Lowry assay. Immediately after homogenization, Complex I activity was measured as the kinetic change in 600 nm absorbance following the addition of 2,6-dichloroindophenol (DCIP) as the terminal electron acceptor and the artificial substrate Coenzyme Q10, resulting in the highly rotenone-sensitive oxidation of NADH [31]. The rotenone insensitive change in 600 nm absorbance was subtracted. Complex IV activity was measured by the kinetic change in absorbance at 550 nm following the addition of cytochrome c. Citrate synthase was measured using a coupled reaction with oxaloacetate, acetyl-CoA, and 5,5-dithiobis-(2,4-nitrobenzoic acid) essentially as described [54]. For all assays, pseudo first-order rate constants were measured and normalized to protein concentration.

### Immunohistochemistry and immunofluorescence

Brains were sectioned in the coronal plane at 30 µm thickness using a freezing microtome. Free-floating brain sections were thoroughly washed in 1X PBS to remove cryoprotectant and then blocked in 1% normal goat serum (NGS) in PBS for 1 h, then incubated with anti-tyrosine hydroxylase (TH) primary antibody (Millipore, cat #: AB152, 1:3000) for ~ 48 h in the same buffer at 4 °C. Next, sections were incubated with biotinylated goat anti-rabbit secondary antibody for 2 h at RT, followed by avidin–biotin peroxidase complex solution (Vector Laboratories ABC Elite) for 2 h at RT. Tissue sections were then developed using DAB chromogen (Vector Laboratories) for ~ 3 min.

For immunofluorescence, tissue sections were incubated in blocking buffer (10% normal donkey serum in PBS with 0.3% Triton-X-100) for 1 h at RT, then incubated for ~ 48 h at 4 °C with primary antibodies (pS129 α-synuclein, BioLegend #825701, 1:10,000) in blocking buffer containing 1% normal goat serum. Following three washes in PBS, sections were incubated with Alexa-conjugated secondary antibodies (AlexaFlour 555-conjugated goat anti-mouse IgG2a) for 2 h at room temperature in blocking buffer containing 1% normal goat serum. Following washing 3 × 5 min in PBS, sections were mounted on microscope slides, air dried overnight and cover slipped using Prolong Diamond antifade mounting medium with DAPI (Life technologies). Low power images were acquired with a Nikon Ti-S epifluorescence microscope using a 20× objective. High power images were acquired with a Leica TCS SP5 laser scanning confocal microscope using a 63× oil objective.

### Stereology

Every 5th section (spanning the SNc) was DAB stained with anti-TH antibody as above. The optical fractionator probe of Stereoinvestigator software (MicroBrightField) was used to obtain an unbiased estimate of TH-positive neurons in the SNc. Contours were drawn around the SNc using a 4× objective. Neurons were counted using a 1.42 NA 60× oil objective with a 50 × 50 µm counting frame and a grid size set to 100 × 100 µm. Only TH-positive cells with a nucleus coming into focus inside the counting frame or touching the two green sides of the frame were counted by an investigator blind to treatment and injected hemisphere.

### Western analysis

Separate injected and uninjected hemispheres of frozen cortex from each animal were dounce homogenized on ice in PBS buffer supplemented with protease and phosphatase inhibitor cocktails (Sigma Aldrich). Following homogenization, sarkosyl was added to 1% and samples were sonicated at 50% amplitude, 1 s on/off for 10 s to shear DNA. Samples were centrifuged at 200,000xg for 60 min at 4 °C in a tabletop centrifuge (Beckman coulter). Supernatants were used for gel electrophoresis as follows: 50 µg protein (measured with BCA assay) from each sample was separated by SDS-PAGE 1 h in a 4–20% mini protean gel (Bio-Rad). Proteins were then transferred onto a 0.2 µM PVDF membrane then blocked in 1:1 LI-COR Odyssey blocking buffer and TBS with 0.05% Tween 20 (TBS-T) for 1 h at RT, then incubated with primary antibody overnight at 4 °C (see Table 1 for detailed list of antibodies used). After washing with TBS-T, membranes were incubated with LI-COR Odyssey secondary antibodies for 2 h at RT and imaged using a LI-COR Odyssey Scanner.

**Table 1:** List of antibodies used for this study

Antibody	Clone	Dilutions	Host species	Company	Cat. No
pS129 alpha synuclein	P-syn/81A	1:10,000 IF	Mouse IgG2a	BioLegend	825,701
Tyrosine hydroxylase	–	1:3,000 IHC	Rabbit	Millipore	AB152
VDAC1	–	1:1,000 WB	Rabbit	Abcam	Ab15895
COX-IV	4D11—B3-E8	1:2,000 WB	Mouse	Cell signaling	11967S
Tomm-20	2F8.1	1:1,000 WB	Mouse	Millipore	MABT166
Ndusf4	EP7832	1:1,000 WB	Rabbit	Abcam	Ab137064
DRP1	D6C7	1:1,000 WB	Rabbit	Cell signaling	8570S
OPA1	D6U6N	1:1,000 WB	Rabbit	Cell signaling	80471S
FIS1	JE40-90	1:500 WB	Rabbit	Novus biologicals	NBP275691
MIRO1	CL1083	1:500 WB	Mouse	Abcam	Ab188029
GAPDH	6C5	1:10,000 WB	Mouse	Millipore	MAB374

### Statistical analysis

GraphPad Prism 8 software was used for statistical analysis and graphing. Data were tested for normality and appropriate parametric tests were performed. For data that failed to show normality, appropriate non-parametric tests were performed, as described in figure legends and Table 2. Data are represented as mean ± SEM.

**Table 2:** Statistical Analyses

Figure	Comparison	Type of test	Statistic	*p* values and 95% CI
3B	3 mo. stereology interaction	Two-Way ANOVA	F_(1,36)_ = 0.6118	*p* = 0.4392
	3 mo. stereology hemisphere	Two-Way ANOVA	F_(1,36)_ = 3.908	*p* = 0.0557
	3 mo. stereology treatment	Two-Way ANOVA	F_(1,36)_ = 6.667	p = 0.0140
	3 mo. Monomer v PFF: Contra	Sidak’s multiple comparisons		95% CI: -619.3 to 2015 *p *= 0.3779
	3 mo. Monomer v PFF: Ipsi	Sidak’s multiple comparisons		95% CI: 26.42 to 2751 *p *= 0.0451
	3 mo. Contra vs Ipsi: Monomer	Sidak’s multiple comparisons		95% CI: -799.1 to 1785 *p *= 0.6145
	3 mo. Contra vs Ipsi: PFF	Sidak’s multiple comparisons		95% CI: -289.8 to 2568 *p *= 0.1370
3C	3 mo.: % uninjected	Unpaired two-tailed t-test	t = 1.808, df = 18	*P *= 0.0874
3E	6 mo. stereology interaction	Two-Way ANOVA	F_(1,42)_ = 2.311	*p *= 0.1359
	6 mo. stereology hemisphere	Two-Way ANOVA	F_(1,42)_ = 6.765	*p *= 0.0128
	6 mo. stereology treatment	Two-Way ANOVA	F_(1,42)_ = 2.692	*p *= 0.1083
	6 mo. Monomer v PFF: Contra	Sidak’s multiple comparisons		95% CI: -724.8 to 780.1 *P *= 0.9954
	6 mo. Monomer v PFF: Ipsi	Sidak’s multiple comparisons		95% CI: -27.25 to 1478 *P *= 0.0606
	6 mo. Contra vs Ipsi: Monomer	Sidak’s multiple comparisons		95% CI: -488.0 to 938.8 *P *= 0.6853
	6 mo. Contra vs Ipsi: PFF	Sidak’s multiple comparisons		95% CI: 176.8 to 1714 *P *= 0.0134
3F	6 mo.: % uninjected	Unpaired two-tailed t-test	t = 2.286, df = 21	*P *= 0.0328
4B	3 mo. VDAC1 Interaction	Two-way ANOVA	F(_1,28)_ = 2.969	*p *= 0.0959
	3 mo. VDAC1 Hemisphere	Two-way ANOVA	F_(1,28)=_ 2.969	*p *= 0.0959
	3 mo. VDAC1 Treatment	Two-way ANOVA	F_(1,28)_ = 6.355	*p *= 0.0177
	3 mo. VDAC1, Ipsi vs Contra: PFF	Sidak’s multiple comparisons		95% CI: -1.045 to -0.01615 *p *= 0.0424
	3 mo. VDAC1, Monomer v PFF: Contra	Sidak’s multiple comparisons		95% CI: -1.168 to -0.1390 *p *= 0.0112
4C	3 mo. COX-IV Interaction	Two-way ANOVA	F_(1,28)_ = 0.1284	*p *= 0.7227
	3 mo. COX-IV Hemisphere	Two-way ANOVA	F_(1,28)_ = 0.1284	*p *= 0.7227
	3 mo. COX-IV Treatment	Two-way ANOVA	F_(1,28)_ = 7.425	*p *= 0.0110
	3 mo. COX-IV, Monomer v PFF: Ipsi	Sidak’s multiple comparisons		95% CI: -2.925 to 0.1175 *p *= 0.0742
	3 mo. COX-IV, Monomer v PFF: Contra	Sidak’s multiple comparisons		95% CI: -2.599 to 0.4438 *p *= 0.1997
4D	3 mo. Tomm-20 Interaction	Two-way ANOVA	F_(1,28)_ = 0.4529	*p *= 0.5065
	3 mo. Tomm-20 Hemisphere	Two-way ANOVA	F_(1,28)_ = 0.4529	*p *= 0.5065
	3 mo. Tomm-20 Treatment	Two-way ANOVA	F_(1,28)=_ = 4.356e-0.005	*p *= 0.9948
4E	3 mo. Ndusf4 Interaction	Two-way ANOVA	F_(1,28)_ = 0.03257	*p *= 0.8581
	3 mo. Ndusf4 Hemisphere	Two-way ANOVA	F_(1,28)_ = 0.03257	*p *= 0.8581
	3 mo. Ndusf4 Treatment	Two-way ANOVA	F_(1,28)_ = 0.01352	*p *= 0.9083
4G	6 mo. VDAC1 Interaction	Two-way ANOVA	F_(1, 32)_ = 2.126	*p *= 0.1546
	6 mo. VDAC1 Hemisphere	Two-way ANOVA	F_(1,32)_ = 2.126	*p *= 0.1546
	6 mo. VDAC1 Treatment	Two-way ANOVA	F_(1,32)_ = 0.1131	*p *= 0.7388
	6 mo. VDAC1, Ipsi vs Contra: PFF	Sidak’s multiple comparisons		95% CI: -0.06415 to 0.9943 *p *= 0.0926
4H	6 mo. COX-IV Interaction	Two-way ANOVA	F_(1,32)_ = 3.299	*p *= 0.0787
	6 mo. COX-IV Hemisphere	Two-way ANOVA	F_(1,32)_ = 3.299	*p *= 0.0787
	6 mo. COX-IV Treatment	Two-way ANOVA	F_(1,32)_ = 0.3907	*p *= 0.5364
	6 mo. COX-IV, Ipsi vs Contra: PFF	Sidak’s multiple comparison		95% CI: 0.04751 to 1.051 *p *= 0.0299
	6 mo. COX-IV, Monomer v PFF: Ipsi	Sidak’s multiple comparison		95% CI: -0.8706 to 0.1326 *P *= 0.1791
4I	6 mo. Tomm-20 Interaction	Two-way ANOVA	F_(1,32)_ = 0.06019	*p *= 0.8078
	6 mo. Tomm-20 Hemisphere	Two-way ANOVA	F_(1,32)_ = 0.06019	*p *= 0.8078
	6 mo. Tomm-20 Treatment	Two-way ANOVA	F_(1,32)_ = 0.3443	*p *= 0.5615
4 J	6 mo. Ndusf4 Interaction	Two-way ANOVA	F_(1,28)_ = 0.02204	*p *= 0.8830
	6 mo. Ndusf4 Hemisphere	Two-way ANOVA	F_(1,28)_ = 0.02204	*p *= 0.8830
	6 mo. Ndusf4 Treatment	Two-way ANOVA	F_(1,28)_ = 0.01168	*p *= 0.9147
5B	3 mo. DRP1 Interaction	Two-way ANOVA	F_(1,28)_ = 0.2058	*p *= 0.6536
	3 mo. DRP1 Hemisphere	Two-way ANOVA	F_(1,28)_ = 0.2058	*p *= 0.6536
	3 mo. DRP1 Treatment	Two-way ANOVA	F_(1,28)_ = 7.005	*p *= 0.0132
	3 mo. DRP1, Monomer vs PFF: Ipsi	Sidak’s multiple comparison		95% CI:-0.01347 to 0.3597 *p *= 0.0723
5C	3 mo. OPA1 Interaction	Two-way ANOVA	F_(1,28)_ = 0.05688	*p *= 0.8132
	3 mo. OPA1 Hemisphere	Two-way ANOVA	F_(1,28)_ = 0.05688	*p *= 0.8132
	3 mo. OPA1 Treatment	Two-way ANOVA	F_(1,28)_ = 2.698	*p *= 0.1116
5D	3 mo. FIS1 Interaction	Two-way ANOVA	F_(1,28)_ = 0.003612	*p *= 0.9525
	3 mo. FIS1 Hemisphere	Two-way ANOVA	F_(1,28)_ = 0.003612	*p *= 0.9525
	3 mo. FIS1 Treatment	Two-way ANOVA	F_(1,28)_ = 0.06165	*p *= 0.8057
5E	3 mo. MIRO Interaction	Two-way ANOVA	F_(1,28)_ = 0.3095	*p *= 0.5824
	3 mo. MIRO Hemisphere	Two-way ANOVA	F_(1,28)_ = 0.3095	*p *= 0.5824
	3 mo. MIRO Treatment	Two-way ANOVA	F_(1,28)_ = 0.009319	*p *= 0.9238
5G	6 mo. DRP1 Interaction	Two-way ANOVA	F_(1,32)_ = 0.2508	*p *= 0.6199
	6 mo. DRP1 Hemisphere	Two-way ANOVA	F_(1,32)_ = 0.2508	*P *= 0.6199
	6 mo. DRP1 Treatment	Two-way ANOVA	F_(1,32)_ = 0.5342	*p *= 0.4702
5H	6 mo. OPA1 Interaction	Two-way ANOVA	F_(1,32)_ = 0.06358	*p *= 0.8025
	6 mo. OPA1 Hemisphere	Two-way ANOVA	F_(1,32)_ = 0.06358	*p *= 0.8025
	6 mo. OPA1 Treatment	Two-way ANOVA	F_(1,32)_ = 0.2102	*p *= 0.6497
5I	6 mo. FIS1 Interaction	Two-way ANOVA	F_(1,32)_ = 0.1527	*P *= 0.6985
	6 mo. FIS1 Hemisphere	Two-way ANOVA	F_(1,32)_ = 0.1527	*P *= 0.6985
	6 mo. FIS1 Treatment	Two-way ANOVA	F_(1,32)_ = 1.472	*P *= 0.2339
5 J	6 mo. MIRO Interaction	Two-way ANOVA	F_(1,32)_ = 0.01677	*p *= 0.8978
	6 mo. MIRO Hemisphere	Two-way ANOVA	F_(1,32)_ = 0.01677	*p *= 0.8978
	6 mo. MIRO Treatment	Two-way ANOVA	F_(1,32)_ = 0.1738	*p *= 0.6795

## Results

### Generation and validation of α-synuclein PFFs

Although we and others have successfully used probe tip sonication of α-synuclein fibrils to generate PFFs that induce α-synuclein aggregation upon intracranial injection of rodents [1, 14, 22, 45, 46, 59], it has been reported that PFFs longer than 50 nm are not as effective at producing pS129-immunoreactive α-synuclein pathology [1, 61]. A major drawback of probe tip sonicators is the heat generated, which limits the length of time that fibrils can be sonicated. Cup horn sonication is typically conducted with the sample submerged in a circulating water bath to remove heat and thereby maintain the sample at a cool constant temperature, which enables longer sonication time. It has previously been shown that 1 h sonication of α-synuclein fibrils in a water bath sonicator yields smaller size PFFs that induce greater α-synuclein pathology compared to sixty 0.5 s pulses with a probe tip sonicator [39]. Here, we used a cup horn sonicator in a circulating water bath to generate α-synuclein PFFs by 1 h sonication of α-synuclein fibrils that were produced by shaking concentrated recombinant monomeric α-synuclein for 7 days (Fig. 1A). Following sonication (Fig. 1B), PFFs were rigorously analyzed using electron microscopy (EM) and dynamic light scattering (DLS) to assess the length and uniformity of the sonicated fibrils. Analysis of fibril lengths in electron micrographs revealed a uniform population of 10–45 nm fibrils with a median length of 29.5 nm (Fig. 1C, D) while DLS analysis estimated a median radius of 13.5 nm (Fig. 1E). Both analyses showed the absence of longer fibril species that can typically be generated using shorter sonication protocols or probe tip sonicators. Following confirmation that our sonication protocol resulted in a relatively homogeneous population of predominantly small (< 30 nm) PFFs, we performed unilateral injections targeting the dorsal striatum. Separate cohorts of mice were sacrificed 3 months and 6 months post injection (p.i.) and brains were harvested for biochemical and neuropathological analyses to determine the extent of α-synuclein aggregation, nigral cell loss and mitochondrial dysfunction (Fig. 1F).

### Sonicated α-synuclein PFFs resulted in widespread pathology and fast fibril maturity

**Fig. 2:**
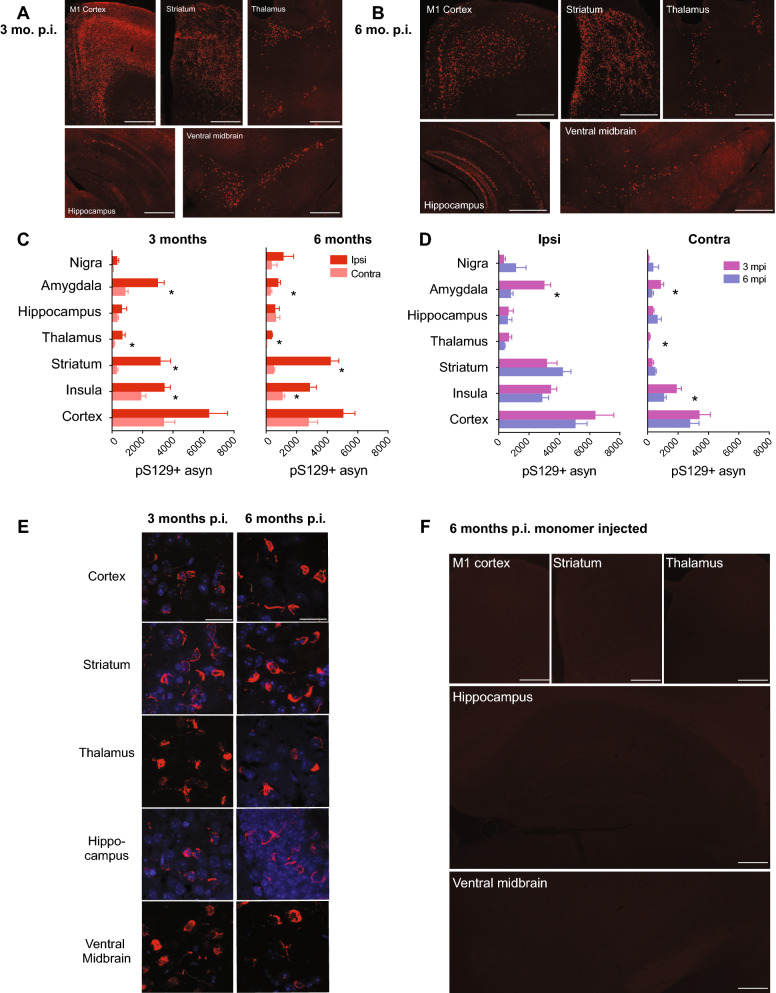
pS129 Immunofluorescence of PFF-injected mice. **A** Representative cortex, striatum, thalamus, hippocampus, and ventral midbrain images of pS129 α-synuclein positive aggregates in PFF-injected mice at 3 months p.i. **B** Representative cortex, striatum, thalamus, hippocampus, and ventral midbrain images of pS129 α-synuclein positive aggregates in PFF-injected mice at 6 months p.i. **C** Number of pS129 α-synuclein aggregates in each brain region measured using Nikon NIS elements software at 3 months (left). Multiple t-test comparison with FDR correction (Q set at 1%) revealed the number of pS129 α-synuclein aggregates was significantly higher in the ipsilateral hemisphere compared to contralateral hemisphere in the amygdala (*p* = 0.0003), thalamus (*p* = 0.0062), striatum (*p* = 0.0015), and insular cortex (*p* = 0.0046) across 3-5 sections from N = 3 PFF-injected mice. 6 months (right), pS129 quantification with Multiple t-tests with FDR correction (Q set at 1%) showed greater pS129 α-synuclein aggregates in the ipsilateral hemisphere compared to contralateral hemisphere in the amygdala (*p* = 0.0099), thalamus (*p* < 0.000001), striatum (*p* = 0.000002), and insular cortex (*p* = 0.000389). **D** The number of pS129 α-synuclein positive aggregates in each region was significantly decreased at 6 months compared to 3 months p.i. in the amygdala (ipsilateral *p* = 0.00365 and contralateral *p* = 0.019), as well as contralateral thalamus and insula cortex (*p* = 0.0378, 0.0170). **E** High resolution confocal imaging of pS129 α-synuclein positive inclusion morphology at 3 and 6 months p.i. in the cortex, striatum, thalamus, hippocampus, and ventral midbrain. **F**. pS129 α-synuclein positive inclusions were detected 6 months post intrastriatal monomer injections. Scale bars are 1000 µm for A, B, and F, and 150 µm for E

We first determined the extent to which the PFFs generated by our 1 h cup horn sonication protocol seeded the formation of α-synuclein aggregates upon intrastriatal injection of mice. We harvested brains from mice 3 months and 6 months after unilateral intrastriatal injection of either α-synuclein PFFs or α-synuclein monomer and analyzed coronal sections by immunofluorescence using an antibody specific for α-synuclein phosphorylated at serine 129 (pS129), which is a well-established marker of pathological synuclein aggregates [19, 24]. In brain sections from mice injected with PFFs, we observed widespread pS129 α-synuclein immunoreactivity, most notably in the motor and insular cortices, striatum, thalamus, hippocampus, amygdala, and ventral midbrain (Fig. 2A, B). At 3 months p.i., pS129 α-synuclein immunoreactivity was consistently greater in the ipsilateral hemisphere compared to the contralateral hemisphere (Fig. 2C, left). The greatest level of immunoreactivity was found in the ipsilateral insular cortex, striatum, and amygdala (Fig. 2C), and indicates that our sonication method yields PFFs that induce robust and widespread pathology in many distinct brain nuclei with projections to the striatum. We observed a similar overall pattern of pS129 α-synuclein immunoreactivity at 6 months p.i. (Fig. 2B, C right). However, compared to 3-month p.i., there was a significant reduction in pS129 α-synuclein immunoreactivity at 6-month p.i. in the amygdala of the ipsilateral hemisphere (Fig. 2D, left) and significant reductions in the insula, thalamus, and amygdala of the contralateral hemisphere (Fig. 2D, right). Contrary to previous studies reporting a significant decrease in level of α-synuclein aggregates in the substantia nigra at 6 months p.i. compared to 3 months p.i. [1], we did not observe significant differences in the level of α-synuclein aggregates in the substantia nigra comparing 3 months to 6 months p.i. in either hemisphere. Qualitative evaluation of pathology in basal ganglia nuclei downstream of the striatum, all of which are affected in PD, shows that intrastriatal injection leads to pathology in the parafascicular nucleus of the thalamus (PFn), and substantia nigra pars reticulata (SNr), which are output nuclei of the basal ganglia (Table 3). No pathology was observed elsewhere in the basal ganglia.

**Table 3:** Presence of pS129-synuclein immunoreactive protein aggregates

Basal Ganglia Nuclei	3 months	6 months
GPe	ND	ND
GPi	ND	ND
PFn	P	P
STN	ND	ND
SNr	P	P

ND = Not detected, P = Present

Live imaging of cortical PFF-induced α-synuclein inclusions has shown that aggregation occurs in stages, progressing from neuritic inclusions to punctate immature somatic inclusions and finally to mature filamentous inclusions surrounding the nucleus [64]. We performed a similar qualitative analysis of pS129 α-synuclein aggregates in images acquired using confocal microscopy. Our analyses revealed that inclusions in the cortices and striatum had the appearance of mature filamentous inclusions at 3 months p.i., while thalamic, hippocampal and nigral inclusions were still in an immature somatic state (Fig. 2E, left column). At 6 months p.i., all brain regions analyzed had the appearance of fully matured somatic inclusions (Fig. 2E, right column). The presence of inclusions with mature appearance as early as 3 months p.i. indicates that our methods generate PFFs capable of corrupting the endogenous α-synuclein to form inclusions that progress to mature filamentous inclusions within 3 months. No pS129 alpha synuclein immunoreactivity was detected in monomer-injected mice (Fig. 2F).

### Loss of TH-immunoreactive cells in the substantia nigra of PFF-injected mice

**Fig. 3:**
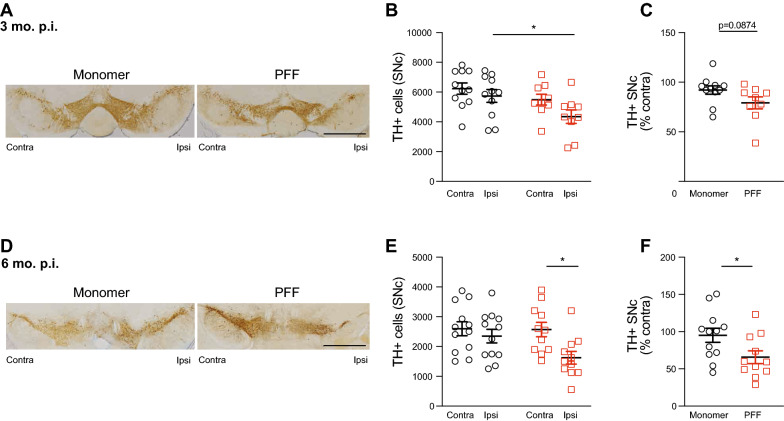
PFF-induced loss of TH+ cells in SNc. **A** Representative TH DAB IHC of monomer and PFF-injected mouse 3-month p.i. **B **TH+ cells in the SNc were counted using unbiased stereology and revealed a significant decrease in the ipsilateral hemisphere of PFF-injected mice compared to ipsilateral monomer (Two-way ANOVA, main effect of PFF, F _(1,36)_ = 6.667, *p* = 0.014; Monomer vs PFF: ipsilateral, Sidak’s multiple comparisons, *p* = 0.0451). **C** No change in TH+ cell counts when normalized as percent of contralateral hemisphere in monomer- and PFF-injected mice, respectively (Unpaired Student’s t-test, *p* = 0.0874, N = 11 monomer, 9 PFF). **D** Representative images TH DAB IHC of monomer and PFF injected mouse at 6 months p.i. **E** PFF-injected mice had a significant reduction in TH+ cells in the ipsilateral hemisphere compared to contralateral, no changes were detected in monomer (Two-way ANOVA, main effect of hemisphere F _(1,42)_ = 26.765, *p* = 0.0128; contralateral vs. ipsilateral: PFF, Sidak’s multiple comparisons *p* = 0.0134, N = 12 monomer, 11 PFF). **F** When normalized as percent of contralateral hemisphere, PFF-injected mice showed significant decrease in TH+ cells compared to monomer-injected mice (Unpaired Student’s t-test, *p* = 0.0328). Scale bars are 200 µm

To determine the extent to which the intrastriatal PFFs induced loss of dopaminergic neurons within the substantia nigra, we used rigorous unbiased stereology to assess the number of tyrosine hydroxylase positive (TH+) cells in coronal sections systematically spanning the substantia nigra. At 3 months p.i., there was a significant decrease in TH immunoreactive cells in the ipsilateral hemisphere of mice injected with PFFs compared to monomer (Fig. 3A, B). No significant difference was detected between monomer and PFF-injected mice when the numbers of TH immunoreactive cells were plotted as a percentage of the contralateral hemisphere (Fig. 3C). At 6 months p.i., PFF-injected mice showed a significant decrease in TH immunoreactive cells in the ipsilateral compared to the contralateral hemisphere (Fig. 3D, E). Additionally, when analyzed as percent of contralateral hemisphere neurons, PFF-injected mice had significantly less TH immunoreactive cells compared to monomer-injected mice (Fig. 3F).

### Mitochondrial protein alterations induced by α-synuclein PFFs

**Fig. 4:**
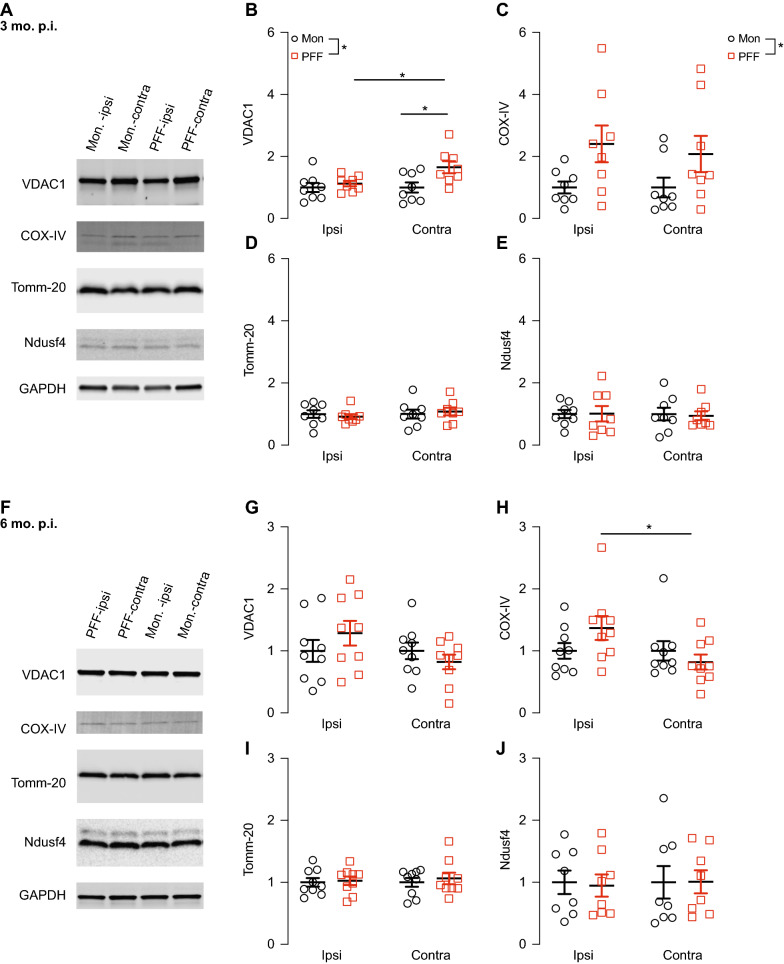
PFF-induced alteration of mitochondrial protein levels. **A** Representative western blot images of mitochondrial proteins VDAC1, COX-IV, Tomm-20, and Ndusf4 in homogenized cortex samples from monomer- and PFF-injected mice 3 months p.i. **B** Increased levels of VDAC1 in the contralateral compared to ipsilateral hemisphere of PFF-injected mice (Two-way ANOVA, main effect of PFF, F _(1,28)_ = 6.355; ipsilateral vs contralateral: PFF, Sidak’s multiple comparison’s *p* = 0.0424; N = 8 monomer, 8 PFF) which was maintained when the contralateral hemispheres were compared between monomer- and PFF-injected mice (Monomer vs PFF: contralateral, Sidak’s multiple comparison’s *p* = 0.112). **C** COX-IV levels showed a main effect of PFF injection (Two-way ANOVA main PFF effect, F _(1,28)_ = 7.425, *p* = 0.0110). No changes were detected in levels of Tomm-20 (**D**) or Ndusf4 (**E**) via Two-way ANOVA or multiple comparisons. **F** Representative western blot images of mitochondrial membrane proteins VDAC1, COX-IV, Tomm-20, and Ndusf4 in monomer- and PFF-injected mice 6 months p.i. **G** No change in VDAC1 levels. **H** COX-IV levels were increased in ipsilateral PFF-injected mice compared to monomer (Two-way ANOVA, Sidak’s multiple comparison’s *p* = 0.0299, N = 9 monomer, 9 PFF). No change detected in Tomm-20 (**I**) or Ndusf4 (**J**) levels. GAPDH was used as a loading/normalization control

α-Synuclein aggregation has been shown to interfere with mitochondrial function [15, 25, 37] and compared to monomeric α-synuclein, PFFs have been found to preferentially bind to mitochondria in primary neurons and decrease respiration capacity [70]. Therefore, we sought to determine the effects of aggregated α-synuclein on mitochondria in the brains of PFF-injected mice. We used western analysis to measure ipsilateral and contralateral cortical levels of the abundant outer mitochondrial membrane proteins VDAC1 and Tomm-20 because α-synuclein is known to bind to these proteins and alter their functions [15, 52]. We also measured the levels of inner mitochondrial membrane protein components of respiratory chain complex I, Ndusf4, and complex IV, cytochrome c oxidase subunit 4 (COX-IV), because complex I and IV protein levels have been found to be reduced in sporadic PD cells [8]. At 3 months p.i. (Fig. 4A), there was a significant increase in VDAC1 protein levels in the contralateral hemisphere of PFF-injected mice compared to the ipsilateral hemisphere of PFF-injected mice (Fig. 4B). There was also a significant increase in VDAC1 protein levels in the contralateral hemisphere of PFF injected mice compared to the contralateral hemisphere of monomer-injected mice (Fig. 4B), suggesting that this effect is specific for the PFF form of α-synuclein. For COX-IV, there was a main effect of treatment in the PFF-injected animals compared to monomer (Fig. 4C). By contrast, the protein levels of Tomm-20 and Ndusf4 were unaffected by injection of monomer or PFFs (Fig. 4D, E). Although the changes in VDAC1 were not detectable at 6 months p.i. (Fig. 4F, G), we observed significantly higher levels of COX-IV in the ipsilateral compared to the contralateral hemisphere of PFF-injected mice (Fig. 4H). Similar to 3 months p.i., no changes were detected in the protein levels of Tomm-20 or Ndusf4 at 6 months p.i. (Fig. 4I, J).

**Fig. 5:**
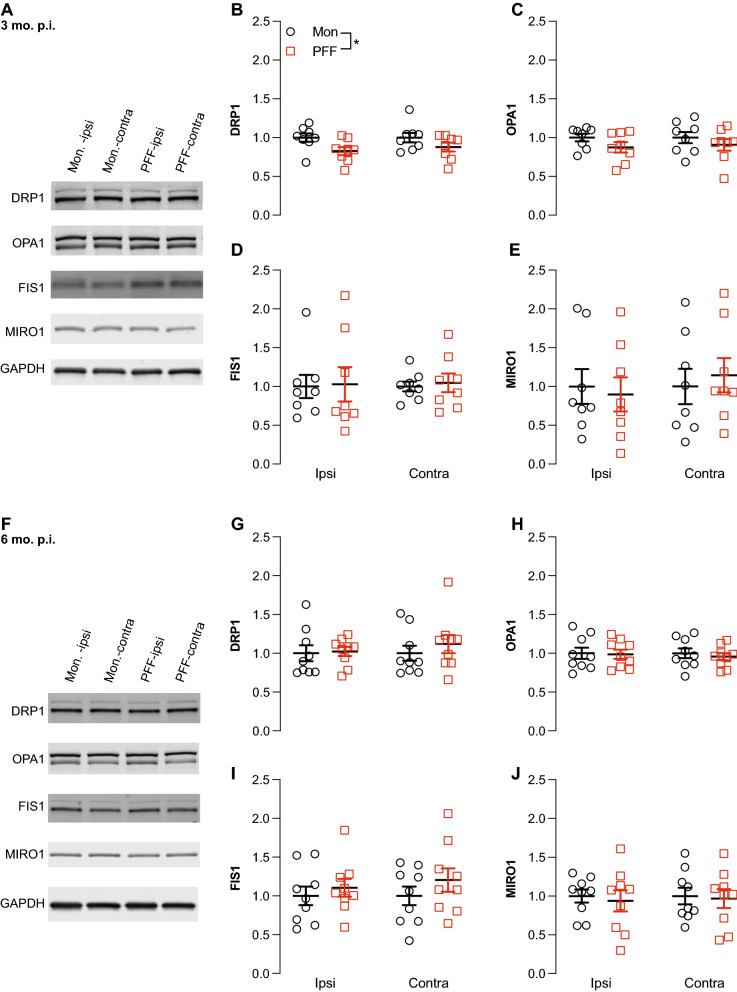
Effect of PFF-seeded pathology on cortical levels of proteins involved in mitochondrial dynamics. **A** Representative western blot images of mitochondrial fusion and fission proteins DRP1, OPA1, FIS1, and MIRO1 in monomer- and PFF-injected mice 3 months p.i. **B** Main effect of PFF injection is observed in DRP1 levels (Two-way ANOVA, F_(1,28)_ = 7.005, *p* = 0.0132, N = 8 monomer, 8 PFF). No changes were detected in levels of OPA1 **(C)**, FIS1 (**D**), or MIRO1 (**E**). **F** Representative western blot images of mitochondrial fusion and fission proteins DRP1, OPA1, FIS1, and MIRO1 in monomer- and PFF-injected mice 6 months p.i. **G–J** No changes in were detected in cortical levels of mitochondrial proteins in monomer or PFF-injected mice 6 months p.i. (N = 9 monomer, 9 PFF). GAPDH was used as a loading/normalization control

We next used western analysis to measure the levels of key proteins mediating mitochondrial dynamics, including the mitochondrial fission proteins DRP1 and FIS1, the mitochondrial fusion and microtubule tethering protein MIRO1, and the inner mitochondrial membrane fusion protein OPA1. At 3 months p.i., two-way ANOVA showed a main effect of PFF injection on the cortical levels of DRP1, consistent with decreased levels of DRP1 in PFF-injected mice compared to monomer-injected mice (Fig. 5B). Although there was a similar trend for the levels of OPA1, two-way ANOVA showed no significant differences between hemispheres or treatments in the cortical levels of OPA1, FIS1, or MIRO1 levels at 3 months p.i. (Fig. 5C–E). At 6 months p.i., there were no detectable differences in the levels of any of these mitochondrial dynamics proteins (Fig. 5F–J).

**Fig. 6:**
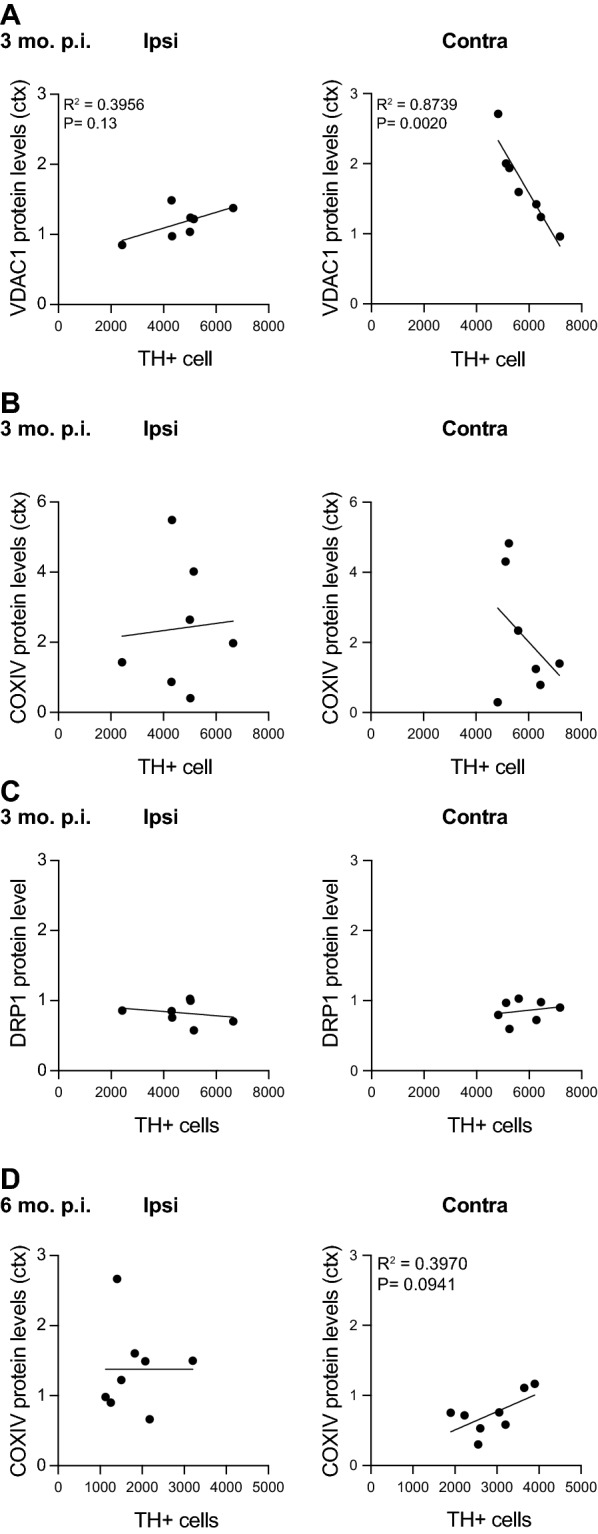
Analysis of PFF-induced TH+ neuron loss correlation with cortical mitochondrial protein levels. **A** Comparison of the number of SNc TH+ neurons determined by stereology to the level of the mitochondrial protein VDAC1 of the ipsilateral (left) and contralateral (right) cortex at 3 months p.i. Simple linear regression analysis revealed a significant correlation of TH+ neuron number and cortical VDAC1 levels in the contralateral hemisphere of PFF-injected mice. **B, C** No correlation was observed with COX-IV or DRP1 cortical protein levels and SNc TH+ cell number in either ipsilateral or contralateral hemisphere. **D** At 6-month p.i., while no significant correlation was observed between cortical COX-IV and SNc TH+ cell number, there was an apparent weak correlation in the contralateral hemisphere (right)

To better understand the relationship between these protein alterations and nigral cell loss, we examined the extent to which the cortical levels of these mitochondrial proteins correlated with the loss of TH+ neurons in the SNc. We observed that cortical VDAC1 levels were negatively correlated with TH+ cell number in the SNc of the contralateral hemisphere at 3 months p.i. (Fig. 6A, right). The levels of cortical VDAC1 were significantly higher as the number of TH+ neurons decreased. No correlation with cell loss was detected in either hemisphere for the protein levels of COX-IV or DRP1 at 3 months p.i. (Fig. 6B, C). For COX-IV at 6-month p.i., while no statistically significant correlation was observed, there was a weak positive correlation, suggesting a parallel decrease with TH+ positive neurons in the SNc. Together, these results indicate that intrastriatal injection of α-synuclein PFFs causes significantly altered cortex levels of key proteins that localize to the inner and outer mitochondrial membranes, as well as proteins that control mitochondrial dynamics, as early as 3 months p.i.

### Aberrant mitochondrial respiratory chain complex function in PFF-injected mice

**Fig. 7:**
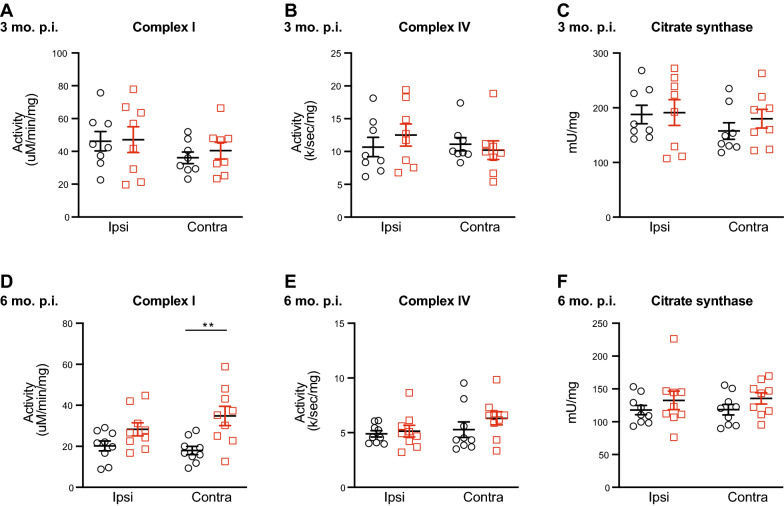
Altered striatal mitochondrial respiratory chain complex activity in PFF-injected mice. Activity of mitochondrial respiratory chain complex I and complex IV, as well as citrate synthase activity, measured at 3 months p.i. (**A–C**) and 6 months p.i. (**D, E**). No significant differences were found at 3 months p.i. (**A–C**). **D** At 6 months p.i., complex I activity was significantly increased in the contralateral hemisphere of PFF-injected animals compared to monomer (Two-way ANOVA, main effect of PFF, F _(1,32)_ = 14.72, *p* = 0.0006; Monomer vs PFF: contralateral, Sidak’s multiple comparison’s test, *p* = 0.0018, N = 9 monomer, 9 PFF). **E, F** There were no significant differences in complex IV or citrate synthase activity at 6 months p.i

To determine the effects of intracranial injection of PFFs and subsequent α-synuclein aggregation on mitochondrial function, we measured the activity of mitochondrial respiratory chain complex I in mitochondria isolated from freshly homogenized striatum. In addition, we measured from frozen aliquots of the same enriched mitochondrial fractions the activity of mitochondrial respiratory chain complex IV as well as citrate synthase, which can be used as a surrogate measure of mitochondrial abundance. At 3 months p.i., there were no significant differences in the specific activity of mitochondrial complex I or complex IV, normalized to protein or to citrate synthase (Fig. 7A–C). However, at 6 months p.i., complex I activity was significantly increased in mitochondria isolated from the contralateral striatum of PFF-injected mice compared to monomer-injected mice (Fig. 7D). Two-way ANOVA revealed a significant main effect of PFF injection on Complex I activity. No changes were detected in complex IV or citrate synthase activity (Fig. 7C, D).

## Discussion

Interactions between pathological forms of α-synuclein and mitochondria have been implicated in synucleinopathies, particularly PD [38]. While studies in vitro and in primary neuronal cultures have provided valuable insights into potential mechanisms by which these interactions drive cellular toxicity, it has been more challenging to study this in vivo. The goal of the current study was to determine the impact of PFF-induced α-synuclein pathology on mitochondrial function, dynamics, and protein homeostasis in vivo*,* using rigorously characterized PFFs that induce robust pS129-α-synuclein aggregates and nigral cell loss upon intrastriatal injection. Our EM and DLS analyses verified that the 1 h continuous cup horn water bath sonication protocol generated small-length PFFs with a relatively uniform size distribution. Although small length PFFs can also be produced using probe tip sonication [1], probe tip sonication often results in loss of material during sonication, production of aerosols that could be hazardous, and heating of the sample, which could denature the protein or otherwise increase variability of the experimental results. Our well-characterized small-length PFFs robustly seeded α-synuclein aggregation in commonly reported brain areas such as the cortices, amygdala, thalamus, and ventral midbrain [22, 35, 44, 46]. We also observed prominent α-synuclein inclusions in the hippocampus (with similar pathology in both ipsilateral and contralateral hemispheres) at both 3 months and 6 months p.i., and more extensive ipsilateral thalamic pathology than typically reported at 3 months p.i. This was somewhat unexpected because, although direct projections from the hippocampus to the ventral striatum are well-known [62], hippocampal projections to the dorsal striatum are not. It is possible that our preparation of shorter PFFs facilitates wider diffusion to the ventral striatum from the site of injection of PFFs in the dorsal striatum, thereby seeding aggregation in hippocampal neurons that project to the ventral striatum. Previous studies have only shown modest thalamic pathology resulting from intrastriatal PFF injection at 90 days p.i. [35], and hippocampal pathology is not typically reported except upon direct intrahippocampal injection in synuclein transgenic mice [53], or 15 months post-intranigral injection of fibrils isolated from DLB patients [40], or upon internal capsule/caudate putamen injection of synuclein transgenic mice [58]. A more recent report showed some hippocampal pathology resulting from bilateral intrastriatal injection of mice with PFFs generated using a 15 °C water bath cup horn sonication protocol similar to the protocol used here and verified to be under 60 nm using DLS [60]. Even in the absence of detectable pS129 α-synuclein immunoreactive inclusions in the hippocampus, proximity ligation assays have revealed abnormal oligomeric forms of α-synuclein in the hippocampus of mice 90 days after intrastriatal injection of PFFs [26].

Our stereological assessment showed a significant decrease of TH+ neurons in the SNc of PFF-injected mice as early as 3 months p.i., consistent with previous reports using relatively short fibrils [22]. Recent biochemical studies have shown that in mixtures of fibril lengths, small α-synuclein fibrils were found to be more seeding competent [1, 61, 63]. Therefore, the robust seeding and spread of α-synuclein aggregates and the robust nigral cell loss induced by intrastriatal injection of the PFFs prepared with our methods is likely attributed to the uniform small (< 30 nm) fibrils produced by our cup horn sonication protocol. While some laboratories have failed to find significant nigral cell loss or α-synuclein aggregation upon intracranial injection of PFFs, best practices should include rigorous characterization of PFFs using EM or DLS to ensure small uniform size PFFs (< 50 nm) prior to intracranial injections.

The primary goal of this study was to determine the impact of PFF-induced α-synuclein inclusions on mitochondrial function, dynamics, and protein homeostasis. We observed alterations in VDAC1, COX-IV, and DRP1 protein levels in the cortex of PFF-injected mice, as well as functional alterations in mitochondrial respiration. Interactions between mitochondria and α-synuclein, particularly aggregated forms, have been implicated in PD pathogenesis [15, 49]. However, the in vivo mechanisms by which these interactions become pathological are not well understood. Therefore, the recapitulation of some features of mitochondrial abnormalities after intrastriatal injection of mice with PFFs is an important step to delineating, and targeting, potential disease-causing mechanisms in vivo*.* While we were able to detect changes in the expression of mitochondrial proteins and in the function of mitochondrial respiratory chain complexes, a previous study did not detect changes in mitochondrial respiration in the striatum and amygdala of mice 5 weeks after intrastriatal injection of PFFs [7]. Notably, we harvested brain tissue for analysis much later (at 3 months and 6 months p.i.). Given the extent of pathology previously observed in rats [1, 13] and mice [22] following longer PFF sonication, and the extent of pathology we observed in the present study compared to previous analyses at 3 months, injection of PFFs generated with our 1 h sonication protocol may elicit detectable changes in mitochondrial respiration not detected in mice injected with PFFs generated using brief probe tip sonication Burtscher, Copin, Sandi and Lashuel [7].

A major challenge to studying mitochondria in vivo stems from the heterogeneity of mitochondrial abundance and function within individual cells (soma, dendrites, or axon terminals), between different types of cells (excitatory/inhibitory neurons, astrocytes, microglia, oligodendrocytes, and endothelial cells), and between brain regions. Thus, analyses using tissue sample homogenates from either the whole brain, or specific brain regions—as we did in the current manuscript—are not direct or sensitive enough to detect localized differences that could be “washed out” when averaging over a large ensemble of mitochondria from different cell types. It remains to be determined whether the significant changes we found in mitochondrial protein levels and mitochondrial enzyme activity levels in PFF-injected brains are driven by neuronal or glial mitochondria. Given the increasing evidence of non-neuronal mitochondrial mechanisms driving neurodegeneration [3], it would be important to define more specifically the cellular location of the mitochondrial abnormalities induced by PFFs. Notably, a recent transcriptomic and neuropathological characterization of PFF-injected mice reveals that PFFs induce microglial-driven neuroinflammation even before the appearance of visible α-synuclein protein inclusions and neurodegeneration [26].

The surprising increase in mitochondrial complex I activity in the non-lesioned hemisphere 6 months post PFF injection suggests that PFF-induced nigral cell loss in the ipsilateral hemisphere causes upregulation of mitochondrial respiration in the contralateral hemisphere. This may correspond to adaptations or sprouting of nigrostriatal synapses in the non-injected hemisphere to maintain overall dopaminergic innervation of the striatum with the progressive loss of ipsilateral nigral neurons. Consistent with this, several groups have reported apparent compensatory upregulation of dopamine and metabolites in the non-lesioned hemisphere of rats after unilateral 6-OHDA lesion [4, 50, 72]. Moreover, comparison of the motor behavior of rats with unilateral and bilateral lesions suggests that the non-lesioned hemisphere can functionally compensate, at least partially, for the loss of dopaminergic innervation in the lesioned hemisphere [51]. The surprising increase in mitochondrial complex I activity that we found in the non-lesioned hemisphere may also be physiologically relevant to human PD, which typically presents with unilateral or non-symmetric motor symptoms for which there is evidence of compensatory mechanisms from the less affected hemisphere [5].

Finally, it is important to note that we and others have previously used the contralateral hemisphere as an internal control or for normalization with the assumption that it is relatively unaffected by the ipsilateral lesion. However, given the significant changes in mitochondrial protein levels and mitochondrial Complex I activity in the contralateral hemisphere, caution should be taken when considering the contralateral hemisphere as an internal control for unilaterally lesioned animal models of PD or other synucleinopathies.

## Conclusions

Mitochondrial dysfunction has been implicated in the pathophysiology of synucleinopathies such as PD, DLB and CBD as well as other neurodegenerative proteinopathies [57, 69]. Our findings establish the utility of the α-synuclein PFF model for studying mechanisms by which α-synuclein pathology drives mitochondrial dysfunction and neurodegeneration. Our findings also support extending the use of the α-synuclein PFF animal model for testing therapeutic approaches aimed at protecting mitochondrial homeostasis and function or lowering α-synuclein aggregation to mitigate PFF-induced nigral DA neuron loss, which is the central neuropathology underlying the motor symptoms of PD.

## Data Availability

Data used for this study is available from the corresponding author upon reasonable request.

## Abbreviations

PD: Parkinson’s disease
pS129: Phospho-serine 129
PBS: Phosphate-buffered saline
PFF: Pre-formed fibril
SNc: Substantia nigra pars compacta
TBS: Tris-buffered saline
TH: Tyrosine hydroxylase
WT: Wild-type
RT: Room temperature
